# Legislating for Madness: Legal Exceptionalism and the Professional Identity of UK Mental Health Nursing. A Discussion Paper

**DOI:** 10.1111/jpm.70182

**Published:** 2026-07-31

**Authors:** Victoria Sweetmore

**Affiliations:** ^1^ University of Derby Derby UK

**Keywords:** legal exceptionalism, Mental Capacity Act, Mental Health Act, mental health nursing, nursing education, professional identity, trauma‐informed care

## Abstract

**Background:**

Mental health nursing in the United Kingdom has developed along a distinctive historical and legal trajectory shaped by statutory frameworks governing mental distress, detention, capacity and coercion. While adult/general nursing largely professionalised through Nightingalean reform, biomedicine and public health, mental health nursing evolved within institutional and legislative systems that positioned nurses within overlapping roles of care, control, advocacy and legal accountability.

**Aim:**

To examine how these historical and legal developments continue to influence contemporary mental health nursing identity, education, workforce preparation and policy and to advance the concept of bounded legal exceptionalism as a framework for understanding the profession's distinctive statutory role.

**Discussion:**

This discussion paper argues that statutory responsibilities remain a significant influence on mental health nursing while recognising that professional identity extends beyond legal powers alone. The concept of bounded legal exceptionalism describes the continuing significance of statutory and coercive frameworks while emphasising that such powers should remain critically scrutinised, rights‐based and tightly circumscribed. International comparisons demonstrate that no single educational model guarantees strong professional identity or workforce preparedness; however, tensions relating to curriculum dilution, specialist preparation and professional visibility remain evident within generic systems.

**Implications for Practice:**

Mental health nursing requires sustained preparation in relational practice, trauma‐informed care, legal literacy and ethical decision‐making. The paper argues that the continuing legal and statutory dimensions of mental health care provide an important rationale for maintaining field‐specific preparation while strengthening supervision, workforce development and rights‐based practice within increasingly integrated healthcare systems.

## Abstract (highlights)

**Accessible Summary:**

- What is Known on the Subject?
  ○ Mental health nursing in the UK has developed differently from adult nursing because it has long been shaped by mental health law, detention and compulsory care.
  ○ Many countries now educate nurses through general nursing programmes, with mental health specialisation happening after qualification
  ○ There are concerns that generic nurse education may reduce the visibility of mental health‐specific knowledge and skills.
- What this Paper Adds to Existing Knowledge?
  ○ This paper introduces the idea of bounded legal exceptionalism to explain how mental health nursing continues to be shaped by law, rights and ethical tensions around care and coercion.
  ○ It shows that mental health nursing identity is influenced not only by education but also by legal systems, workforce structures and professional culture.
  ○ International comparisons show that both specialist and generic systems have strengths and weaknesses, but preparation for mental health practice remains complex
- What are the Implications for Practice?
  ○ Mental health nurses need strong preparation in therapeutic relationships, trauma‐informed care, legal literacy and rights‐based practice.
  ○ Education should balance physical health skills with mental health‐specific knowledge and ethical decision‐making.
  ○ Workforce support, including supervision and reflective practice, is important to reduce burnout and support safe, compassionate care.

**Relevance to Mental Health Nursing:**

This paper is directly relevant to mental health nursing because it examines how legal frameworks, ethical tensions and professional identity continue to shape the field. It explores the ongoing impact of detention, coercion and rights‐based practice on education, workforce preparation and everyday clinical work. By introducing the concept of bounded legal exceptionalism, the paper offers a framework for understanding how mental health nurses can engage critically with statutory responsibilities while maintaining therapeutic, trauma‐informed and recovery‐oriented practice. The paper contributes to current debates about nurse education, workforce sustainability and the future of specialist mental health nursing.

**Key Points:**

- Mental health nursing has developed within a distinct legal and historical context that continues to shape education and practice.
- This paper introduces the concept of bounded legal exceptionalism to explain the continuing influence of statutory responsibilities without reducing professional identity to coercion.
- International comparisons suggest specialist and generic education models each have strengths, but preparation for mental health nursing requires attention to legal, ethical and relational practice.
- The paper argues that field‐specific preparation remains important for supporting safe, rights‐based and recovery‐oriented mental health care.

## 1 Introduction

The professional landscape of nursing in the United Kingdom is characterised by a distinctive split between adult and mental health nursing. While both share a foundation in compassionate, evidence‐based care, they have followed divergent historical, educational and professional trajectories shaped by different legal and institutional contexts.

Unlike adult nursing, which developed through religious and charitable traditions before nineteenth‐century professionalisation (Baly 1995; Dingwall et al. 1988; Hallett 2014), and later through Nightingalean reform, biomedicine and public health (Porter 2002; Scull 2011), mental health nursing emerged within statutory systems of care and control. From nineteenth‐century asylum legislation to the Mental Health Act 1983 (as amended by the Mental Health Act 2007) and the Mental Capacity Act 2005, the governance of ‘madness’ has included powers relating to detention and treatment without consent. These frameworks have shaped mental health nursing practice in ways that differ from adult/general nursing, including responsibilities relating to holding powers, rights information, capacity and risk management. The Nursing and Midwifery Council [NMC] (2018, 2023) sets these expectations across all fields of nursing, but they carry particular significance within mental health nursing, where statutory powers, liberty restriction and coercive practice remain more directly embedded in everyday care.

This paper argues that mental health nursing occupies a historically and legally distinctive position within nursing, shaped by sustained engagement with statutory frameworks governing detention, capacity, coercion and liberty restriction (Abbott 1988; Rose 1999). However, legal exceptionalism is not presented here as the sole basis of mental health nursing identity, nor is coercion understood as its defining feature. Rather, the paper suggests that statutory responsibilities and legal governance remain materially significant dimensions of mental health nursing practice, education and workforce preparation. It further advances the concept of bounded legal exceptionalism, recognising the necessity of some limited coercive powers while arguing these should remain critically scrutinised and tightly circumscribed.

In political theory, exceptionalism denotes the suspension of ordinary legal protections. Schmitt located sovereignty in deciding on the exception (Schmitt 2005), while Agamben (2005) argued exceptional measures can become permanent. These ideas have since been applied to psychiatry, where mental health law has been described as a continuing state of exception (McSherry 2014). Unlike general healthcare law, which is primarily grounded in consent and professional regulation, UK mental health legislation permits detention, treatment and restrictions on liberty in specific circumstances without consent. The Mental Health Act 1983 (as amended) and the Mental Capacity Act 2005 therefore create a distinctive statutory framework for practice. Bartlett and Sandland (2014) describe this as a parallel legal order, while Peay (2011) highlights its entanglement with criminal justice and public protection. Bartlett (2017) further argues that traditional rationales for compulsion, including dangerousness, treatment need and incapacity, are increasingly difficult to sustain, particularly in light of the United Nations Convention on the Rights of Persons with Disabilities (CRPD; United Nations 2006). These tensions continue to shape debates surrounding professional identity, ethics and workforce preparation.

Although the term ‘legal exceptionalism’ is uncommon in nursing scholarship, mental health nursing has historically involved more direct engagement with statutory powers relating to coercion, detention, risk and liberty restriction than most other nursing fields. Contemporary evidence also suggests that educational and regulatory shifts may destabilise aspects of professional identity. A recent cross‐field UK study found that mental health nursing students reported the lowest practice confidence, the greatest barriers to proficiency achievement, and perceived the NMC's generic standards as insufficiently reflecting field‐specific knowledge and skills (Kenwright et al. 2025). These concerns build on earlier critiques that increasingly generic standards risk diluting aspects of mental health nursing identity and specialist preparation (Connell et al. 2022).

This paper: (1) traces the historical genealogy of legal exceptionalism in mental health nursing; (2) examines its manifestations in contemporary practice, education and workforce organisation; (3) situates the UK within an international landscape of field‐based, generic and hybrid registration models; and (4) considers the implications for nursing identity, education and workforce preparation within increasingly integrated systems of care. Drawing on historical, legal, sociological and comparative scholarship, the paper explores how statutory distinctions have contributed to the development of mental health nursing as a distinct field of practice while also considering the limitations and tensions within this framing.

## 2 Genealogy of Legal Exceptionalism

The legal governance of madness in Britain long predates the asylum era. The relationship between law, disorder and care is centuries old and this historical entanglement significantly shaped the development of mental health nursing. As early as the fourteenth century, madness was legally defined and governed. The *Prerogativa Regis* (1324) articulated the monarch's duty of guardianship over the ‘idiot’ (understood as permanently incapable of self‐management) and the ‘lunatic’ (seen as fluctuating in capacity). Although primarily concerned with property management, the statute placed people with mental incapacity under formal legal oversight (Bartlett 1998).

By the sixteenth century, responsibility for care had become tied to poor relief legislation. The Poor Relief Act 1601 (the Elizabethan Poor Law) made parishes responsible for supporting the destitute, including those labelled as ‘mad’. Provision was local and custodial, with individuals housed in poorhouses, workhouses or supported by parish funds. The governance of madness was also linked to concerns about disorder and public safety. The Vagrancy Acts of the seventeenth and eighteenth centuries authorised magistrates to detain ‘lunatics’ found wandering, positioning them alongside beggars and ‘sturdy rogues’. Such legislation criminalised visible madness and reinforced its framing as a threat to social order rather than solely a matter of ill health (Porter 2002).

By the eighteenth century, care for people with mental illness was increasingly commercialised through private madhouses, many of which operated with minimal oversight. The Madhouses Act 1774 introduced the first statutory regulation of these institutions, requiring licensing and admission registers. Although imperfect, the Act reflected growing recognition that madness required distinct forms of governance compared with the voluntary charitable hospitals serving the physically ill (Houston 2014).

The nineteenth century marked a decisive shift. The County Asylums Act 1808 permitted local authorities to establish public asylums, while the Lunacy Act 1845 and County Asylums Act 1845 made their provision compulsory. Together, these Acts created a national system for the confinement and care of people with mental illness, overseen by the Commissioners in Lunacy. Within these institutions, attendants, the forerunners of mental health nurses, were employed primarily as custodians of confinement rather than healers. Their duties were explicitly tied to statutory detention frameworks, marking a divergence from hospital nursing, which during the same period was aligning with Nightingalean reform and the rise of biomedicine (Dingwall et al. 1988; Scull 2011).

Despite moves towards deinstitutionalisation, legal frameworks continued to shape practice. The Mental Treatment Act 1930 introduced voluntary patients but retained coercive powers for those detained against their will. The Mental Health Act 1959 aimed to normalise mental health services within the NHS and reduce stigma, yet statutory detention remained central. Although asylums largely closed from the 1960s onwards, many of the legal and organisational structures underpinning them migrated into general hospitals and community teams (Jones 1972).

Today, the Mental Health Act 1983 (as amended in 2007) and the Mental Capacity Act 2005 continue to shape aspects of contemporary mental health nursing practice. Nurses may exercise holding powers, undertake capacity assessments and ensure patients are informed of their rights (Department of Health and Social Care 2015). Although other nursing fields also engage with legal and ethical decision‐making, mental health nurses remain more directly involved in statutory processes relating to detention, liberty restriction and compulsory treatment.

This genealogy demonstrates that mental health nursing developed through a longstanding relationship between law, institutional care and the governance of mental distress. From medieval guardianship statutes and poor relief legislation to modern mental health and capacity law, nurses have worked within systems shaped by both therapeutic and custodial responsibilities. Legal exceptionalism is therefore not presented here as the sole defining feature of mental health nursing, but as an enduring historical and structural influence on professional identity, statutory responsibilities and educational priorities.

## 3 The Contemporary Professional Split

The asylum era established the roots of legal exceptionalism, but its consequences remain visible in the contemporary divergence between mental health and adult/general nursing. Today, the fields differ not only in educational focus and clinical settings, but also in statutory involvement, interagency relationships and ethical tensions. Mental health nurses remain more directly engaged with statutory frameworks than many other nursing fields. Under the Mental Health Act 1983 (as amended), they may exercise holding powers (Section 5[4]), detain patients pending medical assessment, and ensure detained patients are informed of their rights (Department of Health and Social Care 2015). They also contribute to capacity assessments and Best Interests decision‐making under the Mental Capacity Act 2005. While nurses across all fields engage with legal and ethical frameworks, mental health nursing involves more sustained engagement with detention, coercion, liberty restriction and risk management than is typical within adult nursing practice (NMC 2018, 2023).

The NMC's *Future Nurse: Standards of Proficiency* (NMC 2018, 2023) require all nurses to demonstrate competence in consent, capacity, safeguarding and holistic care. However, mental health nursing has historically prioritised field‐specific competencies such as legal literacy, de‐escalation, trauma‐informed practice and relational security. Concerns have been raised that the *Future Nurse* standards remain heavily weighted towards anatomy, physiology, pharmacology and clinical procedures more closely associated with adult nursing curricula. Scholars argue that competency‐based education can create ‘over‐engineered curricula’ that encourage task‐focused practice and undermine critical autonomy (Collier‐Sewell et al. 2023). Mental health nursing students may therefore experience tensions between meeting broad biomedical and procedural proficiencies while also developing specialist relational, therapeutic and legal competencies (Warrender et al. 2023; Haslam 2023; Fisher 2025).

Critiques of biomedical dominance within mental health nursing are longstanding. Barker (2001) argued that the profession risks losing its relational and person‐centred foundations when distress is understood primarily through biomedical frameworks, emphasising instead narrative, meaning and therapeutic engagement. At the same time, Duxbury (1999) identified biomedical understandings of aggression and risk among both adult and mental health nurses, suggesting these tensions are not unique to mental health nursing alone.

Concerns regarding the dilution of mental health nursing identity also predate the current standards. Critics argue that increasingly generic curricula risk marginalising field‐specific knowledge, therapeutic practice and legal literacy within overcrowded competency frameworks (Connell et al. 2022; Hurley et al. 2008; Warrender et al. 2023, 2024; Haslam 2023). Service‐user perspectives similarly caution that generic approaches may reduce the visibility of lived experience and compassionate care within nurse education (Fisher 2025). However, the relationship between generic education and professional identity remains contested. Australia and New Zealand have retained identifiable mental health nursing cultures despite generic pre‐registration models, suggesting identity is shaped not only by educational structures but also by workforce organisation, professional leadership and post‐registration development.

These debates extend beyond education into organisational and interagency practice. Mental health nurses contribute directly to detention processes, tribunal reports and interagency work involving Approved Mental Health Professionals (AMHPs), police, courts and probation services (Cummins 2010). The case of Valdo Calocane, discharged prematurely before committing three homicides, highlighted failings in risk assessment, communication and interagency planning (Weaver 2024; Care Quality Commission 2024; NHS England 2025). Such cases illustrate the high‐stakes legal and ethical environment within which many mental health nurses practise. Similar pressures are evident within inpatient services, where observation, relational security and risk documentation remain central features of care. Although acute adult services also operate under significant pressures, the statutory management of detention and coercion remains more visible within mental health settings.

These realities are associated with distinctive ethical tensions. While all nurses uphold the principles of autonomy, beneficence, non‐maleficence and justice (Beauchamp and Childress 2019), their application may differ across practice settings. Mental health nurses may be required to balance therapeutic relationships with compulsory treatment, detention or liberty restriction. This creates ethical tensions around power, autonomy and care that are particularly visible within mental health practice, often reflecting a longstanding tension between therapeutic and custodial functions within psychiatric settings (Jones et al. 2024), even though ethical dilemmas also exist across adult and community healthcare settings (Hem et al. 2018). Coercion extends beyond seclusion or restraint to informal and perceived coercion (Paradis‐Gagné et al. 2021). Service‐user accounts consistently describe coercive practices as distressing, dehumanising and damaging to therapeutic trust, with experiences of fear, trauma, powerlessness and disengagement from care frequently reported (Cusack et al. 2018; Wostry et al. 2025). Integrative review evidence further suggests coercive interventions may undermine empathy, trust, engagement and relational safety within therapeutic relationships (Wostry et al. 2025).

More recently, coercion has increasingly been reframed as a human rights concern. Hallett et al. (2024) argue that practices ranging from forced hospitalisation to restraint may threaten autonomy, dignity and recovery, and may conflict with the principles of the United Nations Convention on the Rights of Persons with Disabilities (CRPD). Lived experience literature similarly highlights coercion as pervasive and often traumatising, reinforcing calls for less restrictive and more rights‐based approaches to care. These critiques create an important tension within mental health nursing: the profession remains associated with statutory powers while simultaneously participating in critiques of coercive practice itself.

These tensions also extend into community care. Paradis‐Gagné and Holmes (2022), drawing on Deleuze's concept of societies of control, argue that coercion has increasingly migrated into community treatment orders and surveillance‐oriented forms of practice. Richter (2025) further reviews five commonly cited justifications for coercion, including risk, incapacity, treatment need, protection of others and restoration of autonomy, arguing that none are consistently or ethically sustainable across contexts. This raises difficult questions about how mental health nurses can navigate statutory responsibilities while maintaining therapeutic, recovery‐oriented and rights‐based approaches to care.

In sum, the split between adult and mental health nursing continues to reflect differing historical, legal, organisational and educational trajectories. Mental health nursing remains closely associated with statutory literacy, interagency collaboration and the management of ethical tensions surrounding coercion, risk and autonomy. However, this paper does not argue that mental health nursing is wholly defined by coercion or legal authority alone. Rather, statutory frameworks continue to shape aspects of professional identity, educational preparation and workforce expectations in ways that remain materially significant. These continuing legal and ethical complexities may therefore provide an important rationale for maintaining robust field‐specific preparation within mental health nursing, even within increasingly integrated healthcare systems.

## 4 International Contrasts

International comparisons highlight how unusual the United Kingdom remains in retaining field‐based registration, whereby students qualify directly into adult, child, mental health or learning disability nursing. Globally, the dominant model is generic: the WHO *State of the World's Nursing 2025* report notes that most countries educate nurses through a single RN programme, with mental health incorporated within a generalist curriculum and specialisation occurring post‐registration (World Health Organization [WHO] 2025). Within this context, mental health nursing is frequently underrepresented in workforce planning despite rising global demand for mental health services. Only a limited number of jurisdictions, including Ireland, Malta, South Africa, Japan and parts of Canada, retain distinct psychiatric or mental health nursing pathways. Others, such as Australia and New Zealand, have adopted generic pre‐registration models with postgraduate specialisation. These differing approaches raise questions regarding professional identity, workforce preparation and the place of mental health expertise within contemporary healthcare systems.

Ireland remains one of the clearest comparators to the UK, retaining direct‐entry psychiatric nursing through the Registered Psychiatric Nurse (RPN) pathway regulated by the Nursing and Midwifery Board of Ireland (2025). Irish psychiatric nursing developed within a similar asylum‐based and legislative history to the UK, and contemporary practice continues to involve significant engagement with mental health and capacity legislation, including the Mental Health Act 2001 and the Assisted Decision‐Making (Capacity) Act 2015. Malta similarly retains a direct‐entry psychiatric nursing programme aligned with EU professional standards while maintaining a distinct professional identity within mental health services (Debono 2004; Galea 2019). These models demonstrate the continued viability of field‐specific registration within modern European healthcare systems.

Australia and New Zealand represent a contrasting trajectory. Both countries abolished direct‐entry psychiatric nursing pathways during late twentieth‐century reforms, consolidating education into comprehensive RN programmes with mental health specialisation occurring post‐registration. Critics of these reforms argue that generic programmes may leave graduates underprepared for therapeutic engagement, de‐escalation and rights‐based practice (Happell 2010; Happell and Cutcliffe 2011; Lakeman et al. 2024; Hazelton et al. 2024). Concerns have also been raised regarding the marginalisation of mental health content within broader nursing curricula (Hurley et al. 2024). These debates are longstanding within New Zealand mental health nursing literature, with Prebble (2001, 2008) identifying concerns regarding the invisibility of mental health nursing within comprehensive education models and the implications this may have for professional identity, specialist preparation and workforce development. However, these countries also demonstrate that mental health nursing identities and professional cultures can persist within generic educational systems through post‐registration pathways, professional colleges, workforce structures and continuing professional development. The Australian College of Mental Health Nurses and the College of Mental Health Nurses New Zealand continue to provide strong professional leadership despite the absence of direct‐entry registration pathways.

South Africa and Japan provide further examples of specialist recognition operating within distinct healthcare contexts. South Africa formally recognises psychiatric nursing as a specialist category under the Nursing Act (2005), with competencies emphasising human rights and statutory literacy (South African Nursing Council 2014). However, concerns remain regarding limited clinical exposure and workforce shortages (Heunis et al. 2025; Miller et al. 2025). Japan similarly recognises psychiatric nursing as a specialty, historically shaped by reliance on institutional psychiatric care and increasingly by community‐based services such as psychiatric home‐visit nursing (Japanese Nursing Association 2023; Setoya et al. 2023). In both cases, specialist recognition coexists with wider structural pressures relating to workforce capacity, service reform and models of care.

Canada offers a hybrid model. In western provinces and territories, psychiatric nursing is regulated as a distinct profession with graduates qualifying as RPNs, whereas elsewhere mental health preparation occurs through general RN pathways and post‐registration development (Canadian Institute for Health Information 2023; Registered Psychiatric Nurse Regulators of Canada, n.d.). This creates variation in professional identity, visibility and educational preparation across jurisdictions.

Taken together, these international contrasts suggest that no single model guarantees either strong professional identity or effective workforce preparation. Generic systems may risk reducing the visibility of mental health content within undergraduate education, particularly where post‐registration pathways and professional structures are weak. Equally, field‐specific systems remain shaped by broader pressures relating to workforce shortages, service reform, resource constraints and the ethics of coercion. Nevertheless, the UK's continued commitment to field‐based registration reflects the view that mental health nursing involves sufficiently distinctive legal, relational and therapeutic demands to warrant dedicated educational preparation at the point of registration.

## 5 Counter Arguments and Critical Responses

A common objection to maintaining field‐based mental health nursing is that it reinforces professional divisions at a time when health policy increasingly emphasises integration and parity of esteem between mental and physical health. From this perspective, all nurses should be prepared to care holistically for both body and mind, reducing historical silos and ensuring mental health is embedded across the profession rather than confined to a specialist pathway. Advocates of generic models therefore argue that a single registration route may strengthen parity by ensuring all nurses receive exposure to mental health theory, placements and relational skills during undergraduate education.

Related arguments focus on the sufficiency of generic curricula and competency frameworks. The Nursing and Midwifery Council's *Future Nurse* standards (NMC 2018, 2023) require all nurses to demonstrate competence in communication, safeguarding, consent and person‐centred care. Supporters of generic approaches therefore argue that specialist pre‐registration pathways are unnecessary, particularly where postgraduate education and workplace development can provide additional expertise. Post‐registration specialisation may provide an alternative route to developing mental health expertise where supported by robust professional structures, workforce investment and clearly defined educational pathways (Happell 2010; Happell and Cutcliffe 2011; Nursing Council of New Zealand 2022). This differs somewhat from medical education, where broad undergraduate preparation is followed by prolonged postgraduate specialisation prior to independent specialist practice. In contrast, nursing registration confers earlier field‐specific accountability at the point of qualification, including responsibilities relating to therapeutic engagement, risk management and statutory processes. Concerns nevertheless remain regarding the consistency and accessibility of such preparation within increasingly pressured healthcare systems. International trends are often cited in support of this position, with countries including Australia, New Zealand and the United States producing mental health practitioners through comprehensive RN programmes rather than direct‐entry specialist registration.

A further objection is that the legal dimensions of mental health nursing may be overstated. Critics argue that although mental health legislation provides an important statutory framework, many nursing encounters occur with voluntary service users and rely primarily on therapeutic communication, relational practice and professional judgement rather than formal coercive powers. They further note that legal and ethical decision‐making is not unique to mental health nursing. Adult and community nurses also engage with issues relating to capacity, end‐of‐life care, safeguarding, autonomy and risk, particularly through the Mental Capacity Act 2005 and wider professional accountability frameworks (Scott et al. 2020; Stevens 2013; Regan and Sheehy 2016). From this perspective, legal exceptionalism may risk overstating the distinctiveness of mental health nursing while underestimating shared ethical and legal practice across nursing fields.

These counterarguments warrant serious consideration. Generic educational models may support workforce flexibility and broader mental health awareness across nursing, while international evidence demonstrates that identifiable mental health nursing cultures can persist within generic systems through post‐registration pathways and professional structures. It is also important to acknowledge that mental health nursing cannot be reduced solely to coercion, legislation or statutory authority. Olson (1996), for example, argued that psychiatric nursing has historically struggled to define itself as both ‘fundamental’ to nursing and simultaneously ‘special’ or distinct, warning that professional identity becomes unstable when tied too narrowly to any single defining characteristic. Therapeutic relationships, recovery‐oriented practice, trauma‐informed care, advocacy and relational work therefore remain equally important aspects of the field.

Nevertheless, the argument advanced here is that legal and statutory frameworks continue to shape mental health nursing in ways that remain materially significant for educational preparation and professional identity. Although all nurses engage with ethical and legal decision‐making, mental health nurses remain more directly involved in detention processes, liberty restriction, compulsory treatment, tribunal systems and interagency risk management. These responsibilities require not only legal literacy but also sophisticated relational and ethical skills capable of navigating tensions between care, autonomy, protection and coercion.

Similarly, while generic curricula may provide broad foundations, critics argue that mental health content can become diluted where it competes with increasingly crowded biomedical and procedural requirements (Lakeman et al. 2024; Hurley et al. 2024). Concerns have therefore emerged that generic programmes may not consistently provide sufficient preparation in areas such as de‐escalation, therapeutic engagement, trauma‐informed practice and rights‐based care. However, this does not imply that generic systems inevitably fail, nor that field‐specific registration alone guarantees strong professional identity or workforce preparedness. International evidence instead suggests outcomes are shaped by the interaction between curriculum design, placement quality, professional leadership, workforce investment and post‐registration development.

The role of law within everyday practice is also more complex than either side of the debate sometimes acknowledges. Formal detention may not define every interaction, yet statutory frameworks continue to influence risk assessment, documentation, care planning, safeguarding and accountability structures within mental health services. Coercion may also operate beyond formal detention through informal pressures, perceived coercion and community treatment arrangements (Paradis‐Gagné and Holmes 2022). At the same time, contemporary critiques increasingly question the ethical legitimacy of coercive practices themselves. Richter (2025), for example, argues that many traditional justifications for coercion are difficult to sustain consistently in practice. This creates an important paradox for mental health nursing: the profession remains associated with statutory powers while simultaneously engaging in critical reflection regarding coercion and restriction. Importantly, reducing or abolishing coercive practices would not necessarily erase mental health nursing identity. Distinctive features such as therapeutic engagement, trauma‐informed practice, recovery‐oriented care, relational security and advocacy within complex psychosocial systems extend beyond statutory detention alone. The challenge for contemporary mental health nursing is therefore not whether identity can exist without coercion, but how it can evolve while minimising reliance on coercive frameworks wherever possible.

Taken together, these debates suggest that the question is not simply whether mental health nursing should remain ‘separate’ from general nursing, but how systems can ensure practitioners are adequately prepared for the legal, relational, ethical and organisational realities of mental health practice. The continued retention of field‐based registration within the UK reflects one response to this challenge. Whether this model remains preferable to generic pathways remains a matter of ongoing professional, educational and political debate. However, the enduring statutory dimensions of mental health nursing suggest these questions cannot be reduced solely to workforce flexibility or abstract aspirations towards integration.

## 6 Implications for Education and Workforce

The preceding analysis demonstrates that mental health nursing remains strongly shaped by statutory frameworks, ethical tensions and interagency responsibilities that differ in emphasis from many other nursing fields. The challenge, however, is how to engage critically with this legal distinctiveness without allowing coercion and control to dominate professional identity. The concept of bounded legal exceptionalism provides a useful organising framework by recognising that some statutory powers may be necessary to safeguard individuals in crisis and protect the public, while insisting these powers remain tightly circumscribed, critically scrutinised and not permitted to dominate therapeutic practice. This has important implications for education, workforce development and policy.

First, the analysis highlights the continued importance of field‐specific preparation within mental health nursing. The UK's retention of direct‐entry routes into adult, child, mental health and learning disability nursing has increasingly been debated in light of international trends towards generic registration. Critics argue specialist pathways may reinforce unnecessary professional divisions, while supporters suggest they provide space for developing field‐specific knowledge and skills. The evidence reviewed suggests that mental health nursing requires sustained engagement with de‐escalation, trauma‐informed care, relational practice, legal literacy and rights‐based approaches, which may be more difficult to foreground within heavily generalised curricula. However, field‐specific preparation should not become synonymous with professional insularity. Mental health nursing education must continue evolving in response to integrated care, physical health inequalities and multidisciplinary working. While the *Future Nurse* standards provide a broad foundation, programmes should also prepare students to navigate tensions associated with coercion, autonomy and least restrictive practice. Incorporating lived experience perspectives, survivor scholarship and reflective approaches to ethical decision‐making may strengthen students' understanding of power, rights and therapeutic relationships within mental health care. Questions also remain regarding curriculum balance. Annex B of the NMC standards (NMC 2018, 2023) requires mental health nursing students to demonstrate competence in a range of physical health procedures. While some generic competencies are valuable within integrated healthcare systems, critics argue that excessive emphasis on biomedical and procedural content may risk displacing relational, psychosocial and legal aspects of mental health nursing practice. The issue is therefore how curricula can balance generic and field‐specific preparation within mental health nursing education.

The second implication concerns workforce sustainability. Mental health nursing can be emotionally demanding, particularly where practitioners are required to navigate coercion, power imbalances, risk management and ethical uncertainty. Nurses may be expected to maintain therapeutic relationships while simultaneously implementing restrictions on liberty or participating in compulsory treatment processes. This tension between care and control has long been recognised within mental health nursing literature (Barker 2001; Cummins 2010; Jones et al. 2024) and may contribute to moral distress, burnout and workforce attrition, particularly within increasingly pressured service environments (Haslam 2023). Supporting the workforce requires more than technical or procedural training. Clinical supervision and reflective practice remain important mechanisms through which nurses can process emotional labour, explore ethical dilemmas and sustain recovery‐oriented approaches within high‐pressure environments. Evidence suggests structured supervision may improve job satisfaction, reduce turnover and strengthen professional identity, although access remains inconsistent across services. Strengthening supervision provision may therefore represent an important component of workforce retention and professional support strategies. Retention challenges are also shaped by wider structural pressures, including staffing shortages, increasing service demand, public scrutiny following adverse incidents, and the emotional burden associated with high‐stakes decision‐making. Addressing these pressures requires not only safe staffing and supportive workplaces but also clearer professional development pathways and opportunities for leadership, advanced practice and career progression within mental health services.

Finally, bounded legal exceptionalism also carries implications for policy and service design. While some statutory powers are likely to remain necessary within mental health care, contemporary policy increasingly emphasises reducing coercion and strengthening rights‐based alternatives. Reviews such as the *Independent Review of the Mental Health Act* (2018) have called for greater transparency, accountability and least restrictive practice. For nursing, this implies not only compliance with legal duties but active engagement in developing alternatives to coercive care, including crisis services, peer support, psychologically informed approaches and community‐based interventions. At the same time, where coercive interventions are used, robust oversight and accountability remain essential. The routine collection and scrutiny of data relating to restraint, seclusion and compulsory treatment may help reduce the normalisation of coercion while supporting reflective and ethically informed practice.

These implications resonate with current national and international workforce priorities. In England, the *Fit for the Future: 10‐Year Health Plan* identifies workforce expansion, retention, placement quality and career development as central NHS challenges (Department of Health and Social Care [DHSC] 2025), while also identifying mental health services as a priority area for investment and reform. Similarly, the WHO *State of the World's Nursing 2025* highlights global workforce shortages, educational bottlenecks, uneven placement capacity and concerns regarding nurse wellbeing internationally (WHO 2025). Together, these developments reinforce the importance of ensuring mental health nursing education and workforce planning remain responsive to both contemporary service demands and the distinctive ethical and statutory dimensions of practice.

## 7 Conclusion

This paper has argued that mental health nursing in the UK continues to occupy a historically and legally distinctive position within nursing practice. From medieval guardianship statutes and the asylum era through to the Mental Health Act 1983 and the Mental Capacity Act 2005, the field has developed within statutory frameworks that shape professional responsibilities, ethical tensions and interagency practice in ways that differ from many other nursing contexts. This history has contributed to a professional identity shaped, in part, by the ongoing negotiation of care, autonomy, risk and coercion. International comparisons reinforce the complexity of these debates. Countries retaining specialist pathways, such as Ireland and Malta, demonstrate the continued viability of field‐specific mental health nursing education, while Australia and New Zealand illustrate both the possibilities and tensions associated with generic registration models. The evidence reviewed suggests that generic systems do not inevitably erase professional identity, but they may risk reducing the visibility of mental health content where educational, workforce and professional structures are insufficiently robust. Equally, specialist systems do not automatically resolve wider challenges relating to workforce shortages, curriculum pressures or the ethics of coercion.

The concept of bounded legal exceptionalism offers one way of navigating these tensions. It recognises that statutory powers and legal responsibilities remain important dimensions of mental health nursing practice, while also emphasising that coercion should be minimised, critically scrutinised and never permitted to dominate therapeutic relationships or professional identity. Mental health nursing cannot be reduced solely to law, detention or control; relational practice, advocacy, recovery‐oriented care, trauma‐informed approaches and ethical reflection remain equally central to the field. The analysis presented here suggests that mental health nursing requires sustained preparation in areas including therapeutic engagement, legal literacy, ethical decision‐making, de‐escalation and rights‐based practice. At the same time, curricula must remain responsive to integrated healthcare systems and the growing interdependence of physical and mental health care. Workforce strategies similarly require attention not only to recruitment and retention, but also to supervision, reflective practice, emotional labour and professional support.

Ultimately, this paper does not argue that mental health nursing should remain distinct simply because it is legally exceptional. Rather, it suggests that the enduring statutory, ethical, relational and organisational dimensions of mental health practice continue to create distinctive educational and professional demands that remain materially significant within contemporary healthcare systems. Whether field‐based registration remains the best mechanism for meeting these demands will continue to be debated. However, the persistence of these complexities indicates that discussions regarding the future of mental health nursing education cannot be reduced solely to questions of generic workforce flexibility or organisational integration.

## Conflicts of Interest

The author declares no conflicts of interest.

## Data Availability

The author has nothing to report.
